# Cone-beam computed tomography evaluation of shaping ability of kedo-S square and fanta AF™ baby rotary files compared to manual K-files in root canal preparation of primary anterior teeth

**DOI:** 10.1007/s00784-024-05726-y

**Published:** 2024-05-27

**Authors:** Shaimaa S. El-Desouky, Bassem N. El Fahl, Ibrahim A. Kabbash, Shimaa M. Hadwa

**Affiliations:** 1 Pediatric Dentistry, Preventive Dentistry Department, Faculty of Dentistry, Oral Health, Tanta University, Tanta, Egypt; 2 Oral Medicine, Periodontology, Oral Diagnosis, and Radiology Department, Faculty of Dentistry, Tanta University, Tanta, Egypt; 3 Public Health & Community Medicine Department, Faculty of Medicine, Tanta University, Tanta, Egypt

**Keywords:** Kedo-S square file, Fanta AF™ Baby file, Manual K-file, Primary teeth, Root canal treatment, Cone-beam computed tomography

## Abstract

**Background:**

Pediatric rotary file systems were developed to solve manual file limitations. With many systems available, it may be tricky to select the most appropriate one.

**Aim:**

to assess & compare Kedo-S Square, Fanta-AF™-Baby rotary files with manual K-file concerning removed dentin amount, canal transportation, centric ability & root canal taper using CBCT in primary anterior teeth.

**Design:**

Extracted Seventy-five upper primary anterior teeth with intact 2/3 root length were collected and divided into three groups based on root canal instrumentation, group-I: prepared using K-file, group-II: prepared using Kedo-S Square, and group-III: prepared using Fanta AF™ Baby file. The teeth were imaged with CBCT before & following canal instrumentation. Then, the removed dentin amount was calculated at each root-canal level. The Kruskal-Wallis test was utilized to statistically analyze study data.

**Result:**

The difference among the three groups was highly statistically significant at cervical & apical thirds concerning dentin thickness changes on both mesial & distal sides following canal preparation with the least removed dentin in the Kedo-S Square group(*P* < 0.0001). Regarding transportation & centering ability, a non-significant difference between the three groups was found. 80% of the Fanta AF™ Baby group had good-tapered preparation compared to the Kedo-S Square (72%) and K-file (40%) groups(*P* < 0.05).

**Conclusion:**

Kedo-S Square was preferable to Fanta-AF^TM^-Baby & manual K-files in primary root canal preparation.

## Introduction

Preserving primary teeth till physiological exfoliation enhances mastication, aesthetics, pronunciation, and safeguards children from harmful oral habits [1]. The progressive dental caries in primary teeth, if not treated, can result in rapid pulpal damage [2]. Pulpectomy is the preferred therapy to treat symptomatic primary teeth with chronic radicular pulp inflammation or necrosis; its success is dependent on debris elimination, shaping the root canals to provide a path for irrigants and appropriate obturation materials, and maintaining the uniformity of root anatomy [3]. It is regarded as a challenging technique due to the short, thin ribbon-shaped primary root canals, the root apex’s dynamic changes, and the closeness to the permanent tooth bud [4]. Moreover, it is a time-consuming treatment with behavioral management difficulties [5].

Hand files are frequently utilized for primary teeth pulpectomy however they have some drawbacks as lengthy procedures and iatrogenic errors including the potential for ledge creation, canal transportation, and file breakage [5, 6] so, rotary file systems were introduced to overcome these shortcomings. In 2000, Barr et al. [7] used Profile 0.04 rotary system designed for permanent teeth, in pediatric root canal therapy. Also, ProTaper, Hero 642, K3, Mtwo, FlexMaster, and Wave One, have been used for pediatric endodontics; despite the effectiveness of these systems, their highly tapered lengthy files were challenging for dentists to use in primary teeth [8]. Moreover, lateral canal perforations are possible, particularly in curved primary roots [9]. It thus mandated the invention of a unique pediatric rotary file system.

The Kedo-S Square file is the first exclusively single pediatric rotary file system, which consists of two files, one for the anterior primary teeth (A1) and the other for the posterior primary teeth (P1) with a variably-variable taper [5]. The A1-Kedo-S Square file has a 0.38 mm tip diameter and is color-marked with green and black bands on its handle [5]. Another lately introduced pediatric rotary file is Fanta AF™ Baby file which is made of specially heat-treated wire AF wire (AF^TM^-H) with superior mechanical properties and incredibly excellent resistance to frequent fatigue [10]. Pediatric rotary file systems are supposed to minimize debris extrusion, facilitate root canal obturation, provide consistent shaping and filling, and safeguard the original root canal anatomy [11, 12].

The shaping ability of endodontic instruments has been conventionally assessed by destructive approaches such as Indian ink injection [12] and serial histological sectioning [13]. Micro-computed tomography (micro-CT) is a reference method of analyzing root canal preparation with the advantages of being a reproducible, non-invasive, and non-destructive technique [14]. However, it has the major disadvantage of not being suitable for clinical use and can only be used in laboratory-based studies [15]. Cone beam computed tomography (CBCT) overcomes some of the disadvantages of micro-CT such as higher radiation dose, a longer scanning time, a critical hardware setup, and high cost [16]. The smallest field of view (FOV) option of CBCT allows higher resolution and a lower radiation dose while providing a smaller volume of data to be interpreted [17]. Additionally, the availability of specialized software and programming for interactive visualization of CBCT data has enabled the evaluation of the whole volume of the scanned structure, along with axial, sagittal, and coronal bi-dimensional sections simultaneously [18]. Furthermore, CBCT is more accessible to many researchers and is appropriate for patient care also, it may be as precise as micro-CT in assessing the morphological characteristics of extracted human teeth, demonstrating to be a reliable and non-invasive measurement tool to examine in detail the root canal system morphology [19].

One of the most important rules for root canal shaping is to preserve the original canal anatomy and maintain the maximum dentin thickness by regular preparation to avoid ledging, zipping, perforations, and canal transportation [20]. So, the objective of this research was to compare the shaping ability of hand K-files with Kedo-S Square & Fanta AF™ Baby files by measuring the removed dentin amount, root canal transportation, centering ability, and root canal taper using CBCT in primary anterior teeth. The null hypothesis (H_0_) assumed that there was no difference in the removed dentin amount at the apical, middle, and cervical thirds of upper primary anterior root canals after preparation with hand K-files and two new pediatric rotary files (Kedo-S Square and Fanta AF™ Baby).

## Materials and methods

### Ethical standards and study setting

A comparative in-vitro study was carried out at the Pediatric Dentistry Department, Faculty of Dentistry, Tanta University, and the cone beam x-ray was done at the Oral Medicine and Periodontology, Oral Diagnosis & Oral Radiology Department, Faculty of Dentistry, Tanta University after obtaining the approval of the ethical committee (REC), Faculty of Dentistry, Tanta university, code (#R-PED-9-22-3). Informed written consent from parents was attained to use their children’s extracted teeth in the research.

### Eligibility criteria

Seventy-five upper primary anterior teeth were gathered from the pediatric dentistry department’s outpatient clinic, Faculty of Dentistry, Tanta University which were extracted due to ectopic eruption of permanent successors, serial extraction, infection, or excessive caries with an intact two third of the root length. The inclusion criteria were single-rooted upper primary anterior teeth with no evidence of fractures, cracks, or calcified canals. Teeth with multiple root canals or that had previously undergone root canal therapy or pathologic root resorption comprising over a third of the root had been precluded. Pre-operative mesiodistal and buccolingual periapical radiographs of each selected tooth were first taken to visualize the root canals and determine their length, any fracture, and /or canal calcification.

### Sample size calculation and randomization

The sample size and power analysis were determined using the Epi-Info software statistical package, version 2002, developed by the World Health Organization and the Center for Disease Control and Prevention in Atlanta, Georgia, USA. Based on the previous study results conducted by Waly et al. [21] 80% study power & an alpha (α) level of 0.05, the estimated minimum sample size (n) was a total of 75 teeth, 25 teeth for each group.

Random Allocation Software was used for randomization (Sealed Envelope Ltd. 2021 (https://www.sealedenvelope.com/simple-randomiser/v1/lists)). The sample teeth were randomly selected using a 1:1:1 allocation ratio and a block size of six. An independent person created a computer-generated randomized list, which had been preserved in an ambiguous closed wrapper, to assign teeth that met the inclusion requirements to either of the three groups.

### Teeth grouping

Seventy-five teeth specimens were randomly divided (25 specimens/group) according to different root canal instrumentation:

- **Group-I (positive control group)** (*n* = 25): teeth root canals were prepared using hand K-files (Mani Inc., Japan).
- **Group-II (experimental group)** (*n* = 25): teeth root canals were prepared using A1-Kedo-S Square (Reeganz Dental Care Pvt. Ltd. India) rotary file.
- **Group-III (experimental group)** (*n* = 25): teeth root canals were prepared using Fanta AF^™^ Baby (Shanghai Fanta Dental Materials, SUNGO Certification Company Limited, London, England) rotary file.

### Pre-instrumentation CBCT imaging

Selected teeth were scrubbed of soft tissues and calculus with a hand scaler, cleaned under running water, disinfected with 0.5% sodium hypochlorite (Clorox Co., 10th of Ramadan, Egypt), and kept in sterile saline solution at room temperature which was changed every 24 h until use within three months after extraction. Firstly, three fine notches were done mesiodistally on the root buccal surface of each tooth sample at 3, 5, and 7 mm from the apex denoting the apical, middle, and coronal thirds respectively to standardize all measurements at these three reference points. Then, each tooth sample was separately wax wrapped (Perfect Wax Base Plate, Turkey), then arranged in a linear pattern in a custom-made block of cork designed for each group with a dimension smaller than the CBCT ‘s field of view (FOV). To guarantee specimen standardization for CBCT imaging, each tooth sample was put with its long axis parallel to the long axis of the cork block. Pre-instrumentation CBCT images were done for the three study groups. The used CBCT unit was KaVo OP 3D Vision (Kavo Dental, Biberach, Germany) with the following standards: a kilo voltage of 120 kVP, 5 mA, 80 mm × 80 mm field of view (FOV), 0.125 mm voxel size, an exposure time of 7.4 seconds and a scanning time of 26.9 seconds.

### Root canal instrumentation

After pre-instrumentation CBCT imaging, root canal preparations were done by a single highly trained operator for each group to avoid bias. Firstly, No. 4 round bur was used on a high-speed handpiece under profuse water cooling for removing the enamel and dentin layer as well as superficial caries. Then, access opening was done with a No.330 carbide bur (Mani Inc., Japan,) and root canals were explored for patency with #10 K-file also, to establish a glide path before instrumentation. The working length was estimated using a using #15 K-file presented into the root canal till the file tip was brought out through the apical foramen after that 1 mm was subtracted from the measured length. After that, the root canals were washed with normal saline.

In group-I, root canal preparation was done using 21 mm stainless steel hand K-files (0.02 taper) in quarter pull-turn motion starting with file #15 up to file # 40 reaching the working length [22].

While, in group-II, root canal preparation was started with manual preparation with # 20 K-file before introducing a 16 mm A1-Kedo-S Square rotary file. The A1-file was used in a lateral brushing motion 1–2 times per tooth until the whole working length was reached according to the manufacturer’s specifications using an Endo-Mate DT endodontic motor (NSK, Tokyo, Japan) at 300 rpm and 2.2 N/cm torque [23].

Concerning group-III, manual preparation with # 20 K-file was done before introducing the rotary files. Then, a 16 mm Fanta AF™ Baby rotary file was used to prepare the root canal according to the manufacturer’s specifications using an Endo-Mate DT endodontic motor at 350 rpm and 2 N/cm torque. Four files were used sequentially in the following order: open file #17/0.08, #20/0.04 yellow, #25/0.04 red, and #30/0.04 blue [10]. The coronal third was prepared for straight-line access using the orifice opener, then negotiate the canal with #10 K-file in a watch-wind motion to full working length, to get a patent canal pathway. After that, the other three files were used in a pecking motion to the whole working length.

To keep root canal instrumentation uniform, every hand or rotary file was used on up to 5 teeth and then replaced. Ethylenediaminetetraacetic acid (EDTA) gel (17%) (Meta Biomed Co. Ltd, Chungbuk, Korea) was applied to lubricate files at all times during biomechanical preparation [6]. For all groups, during every file change, irrigation was standardized to 5 ml of 1% sodium hypochlorite, followed by normal saline using a 23-gauge needle throughout the instrumentation procedure. In addition, a #10 K-file was applied to check canal patency after each file removal. Lastly, each root canal was dried with sterile paper points (Dentsply Maillefer, OK, USA).

### Post-instrumentation CBCT imaging

After instrumentation, the teeth samples were repositioned in the same preconstructed blocks with the same orientation as the pre-instrumentation scan then, post-instrumentation CBCT images were taken using the same parameters. On-Demand 3D software was used to analyze all pre- and post-instrumentation images. Its characteristics are version 1.0 (build 1.0.10.7462),× 64 Edition, copyright 2004–2017 Cybermed, Korea and license key 670,094,709.

To compare pre- and post-instrumentation CBCT images with standardized measures, the prepared block for each group was placed in the CBCT’s tray with the positioning laser light accurately adapted without any change in the mesiodistal and buccolingual alignment. Also, measurements were taken at the three standardized reference points at 3, 5, and 7 mm from the apex that refer to the apical, middle, and coronal thirds respectively. Moreover, in the post-instrumentation CBCT image, the same slice number on the cut sections was selected for postoperative reading as the pre-operative one.

### Assessment of removed dentin amount

Quantitative measurements of the dentin thickness taken from the CBCT axial cuts in mesial & distal directions were used to calculate the amount of removed dentin at the three predefined levels (cervical, middle & apical thirds) pre- and post-preparation. These numerical readings had been established: M1 and M2 represent the least distance between the outermost of the root’s mesial portion & mesial wall of the non-instrumented & instrumented canal respectively, Likewise, D1 and D2 stand for the least distance between the outermost of the root’s distal portion & the distal wall of the non-instrumented & instrumented canal respectively [9]. A formula (M1-M2) is used to measure the mesial side removed dentin while (D1-D2) is used to measure the distal side removed dentin.

### Canal transportation and centering ability

The root canal transportation was calculated in the mesiodistal dimension using the formula: (M1 − M2)−(D1 − D2) [10]. If a result value equals 0 (zero), it means there is no transportation, a negative value means there is distal transportation while a positive value means there is mesial transportation.

The ratio of centering ability had been determined by dividing the value of (M1 − M2) by the value of (D1 − D2) or (D1 − D2) / (M1 − M2). A result value of 1.0 indicates total centralization. Once the value became near zero, it meant that the file had a lesser ability to keep itself in the canal’s center [24].

### Root canal taper

Root canal taper was measured as the maximum mesiodistal diameter of the root canal at the three predefined levels (cervical, middle & apical thirds) from the sagittal cut of the post-instrumentation CBCT images. Using the OnDemand software, the root canal preparation was evaluated as good or poor taper. A good taper was described as a progressive reduction from the coronal, middle, to the root canal apical third. When measurements from the coronal, middle, toward root canal apical third, were the same or increased, this indicated a poor taper [25].

Intra-examiner reliability was assessed for five teeth used in the pilot trial, in which CBCT radiographic analysis for all parameters was repeated by the same highly skilled blinded radiologist after a two-week washout period. Excellent agreement was demonstrated by the intraclass correlation coefficient (ICC) value (ICC = 0.92).

### Statistical analysis

The IBM SPSS version 19 (Statistical Package for Social Studies) produced by IBM, Illinois, Chicago, USA was used to arrange, tabulate, and statistically analyze the gathered data. The range, mean, and standard deviations for numerical values were computed. The difference among treatment groups was done using the Kruskal-Wallis test, as the data was not normally distributed. Mann-Whitney test had been applied for pair-wise comparison between every two groups once the Kruskal-Wallis test was significant. The number and percentage were calculated for root canal transportation, centering ability, and root canal taper then the difference among groups was evaluated using the Chi-square test. The significance level was set at *p* < 0.05.

## Results

**Fig. 1:**
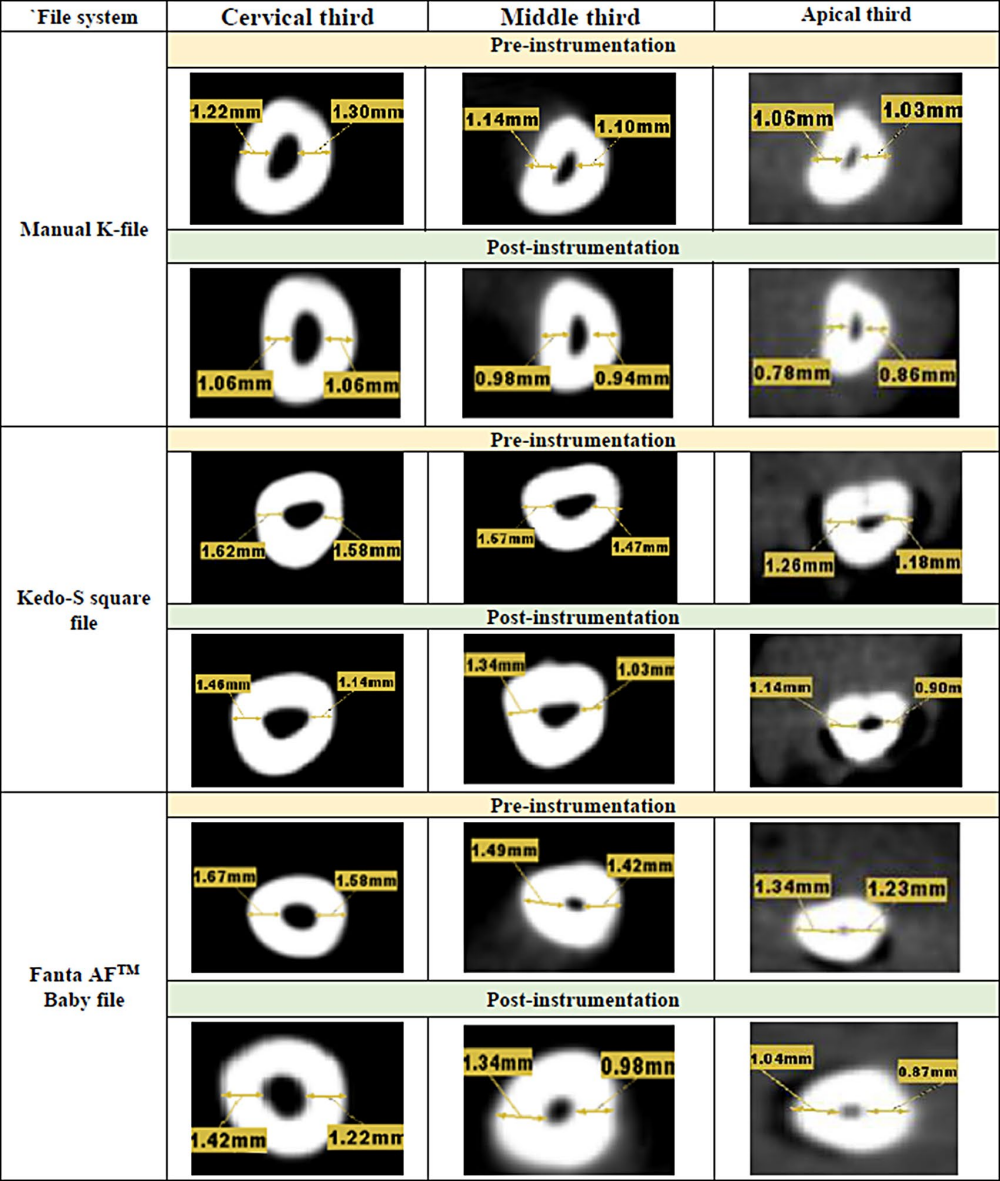
Axial cut of CBCT images showing the amount of dentin thickness pre- and post-instrumentation with manual K-file, Kedo-S Square file, and Fanta AF^™^ Baby file at cervical, middle, and apical thirds

There was a highly statistically significant difference in the amount of removed dentin among the three groups at the cervical & apical thirds on both mesial & distal sides of the root canal with the least removed dentin in the Kedo-S Square group (*P* < 0.0001). The highest amount of removed dentin at the middle third was reported in the manual K-file group (0.13 mm at the mesial side & 0.14 mm at the distal side) as shown in Tables (1 & 2) and Fig. 1. Using pairwise comparison, there was a statistically significant difference between the manual K-file and Kedo-S Square files at different root canal levels in the amount of removed dentin on mesial & distal sides (*P* < 0.05). Moreover, there was a statistically significant difference between the K-file and the Fanta AF^™^ Baby file in the middle third only (*P* < 0.001). While the difference between Kedo-S Square and Fanta AF^™^ Baby files was statistically significant at the cervical & apical thirds (*P* < 0.001).

Regarding root canal transportation, a non-significant statistical difference was found among the three groups at different root canal levels(*P* > 0.05). 36%, 28%, and 40% of the teeth prepared with Kedo-S Square files showed no transportation at the cervical, middle & apical thirds respectively (Table 3). Eight teeth specimens (32%) of the Fanta AF^™^ Baby group revealed no transportation at the middle third compared to four teeth (16%) of the K-file group. The highest mesial transportation in the Fanta AF^™^ Baby group was found at the apical third (56%) while the highest distal transportation in this group was at the cervical third (40%).

**Table 1:** Comparison of the mean & standard deviation (SD) of removed dentin amount between the different study groups on the mesial side at cervical, middle, and apical levels

Root canal level		Manual K-file (Group I)	Kedo-S square (Group II)	Fanta AF^™^ Baby (Group III)	Kruskal-wallis test	p -value	p_1_-value	p_2_-value	p_3_-value
Cervical third	Range	0.13–0.25	0.03–0.18	0.12–0.41	43.627	< 0.001*	< 0.001*	0.733	< 0.001*
	Mean *±* SD	0.188 *±* 0.04	0.075 *±* 0.04	0.210 *±* 0.08					
	Median	0. 18	0.07	0.18					
Middle third	Range	0.07–0.21	0.01–0.15	0.03–0.27	33.417	0.158	< 0.001*	< 0.001*	0.097
	Mean *±* SD	0.133 *±* 0.04	0.057 *±* 0.03	0.080 *±* 0.05					
	Median	0.13	0.05	0.06					
Apical third	Range	0.04–0.15	0.02–0.13	0.07–0.31	21.286	< 0.001*	0.004*	0.093	< 0.001*
	Mean *±* SD	0.086 *±* 0.03	0.057 *±* 0.03	0.112 *±* 0.06					
	Median	0.08	0.06	0.10					

SD: Standard Deviation

*Statistically significant difference at p-value < 0.05

P_1_-value: pairwise comparison between group I and group II

P_2_-value: pairwise comparison between group I and group III

P_3_-value: pairwise comparison between group II and group III

**Table 2:** Comparison of the mean & standard deviation (SD) of removed dentin amount between the different study groups on the distal side at cervical, middle, and apical levels

Root canal level		Manual K-file (Group I)	Kedo-S square (Group II)	Fanta AF^™^ Baby (Group III)	Kruskal-Wallis test	p-value	p_1_-value	p_2_-value	p_3_-value
Cervical third	Range	0.10–0.27	0.01–0.14	0.14–0.40	49.211	< 0.001*	< 0.001*	0.977	< 0.001*
	Mean *±* SD	0.190 *±* 0.04	0.069 *±* 0.03	0.208 *±* 0.08					
	Median	0.19	0.07	0.18					
Middle third	Range	0.06–0.22	0.01–0.12	0.03–0.27	21.26	0.345	< 0.001*	< 0.001*	0.208
	Mean *±* SD	0.144 *±* 0.04	0.056 *±* 0.03	0.078 *±* 0.05					
	Median	0.14	0.06	0.06					
Apical third	Range	0.03–0.16	0.01–0.12	0.04–0.35	17.224	< 0.001*	0.003*	0.418	< 0.001*
	Mean *±* SD	0.088 *±* 0.03	0.057 *±* 0.03	0.106 *±* 0.06					
	Median	0.08	0.06	0.09					

SD: Standard Deviation

*Statistically significant difference at p-value < 0.05

P_1_-value: pairwise comparison between group I and group II

P_2_-value: pairwise comparison between group I and group III

P_3_-value: pairwise comparison between group II and group III

**Table 3:** Comparison of the direction of root canal transportation between the different study groups at the cervical, middle, and apical levels

Root canal level		Manual K-file (Group I)		Kedo-S Square (Group II)		Fanta AF^™^ Baby (Group III)		*X* ^*2*^	p-value
		n	%	n	%	n	%		
Cervical third	No transportation	5	20.0	9	36.0	7	28.0	2.148	0.708
	Mesial Transportation	9	36.0	9	36.0	8	32.0		
	Distal Transportation	11	44.0	7	28.0	10	40.0		
Middle third	No transportation	4	16.0	7	28.0	8	32.0	2.154	0.707
	Mesial Transportation	10	40.0	10	40.0	8	32.0		
	Distal Transportation	11	44.0	8	32.0	9	36.0		
Apical third	No transportation	7	28.0	10	40.0	4	16.0	8.431	0.077
	Mesial Transportation	6	24.0	9	36.0	14	56.0		
	Distal Transportation	12	48.0	6	24.0	7	28.0		

**Fig. 2:**
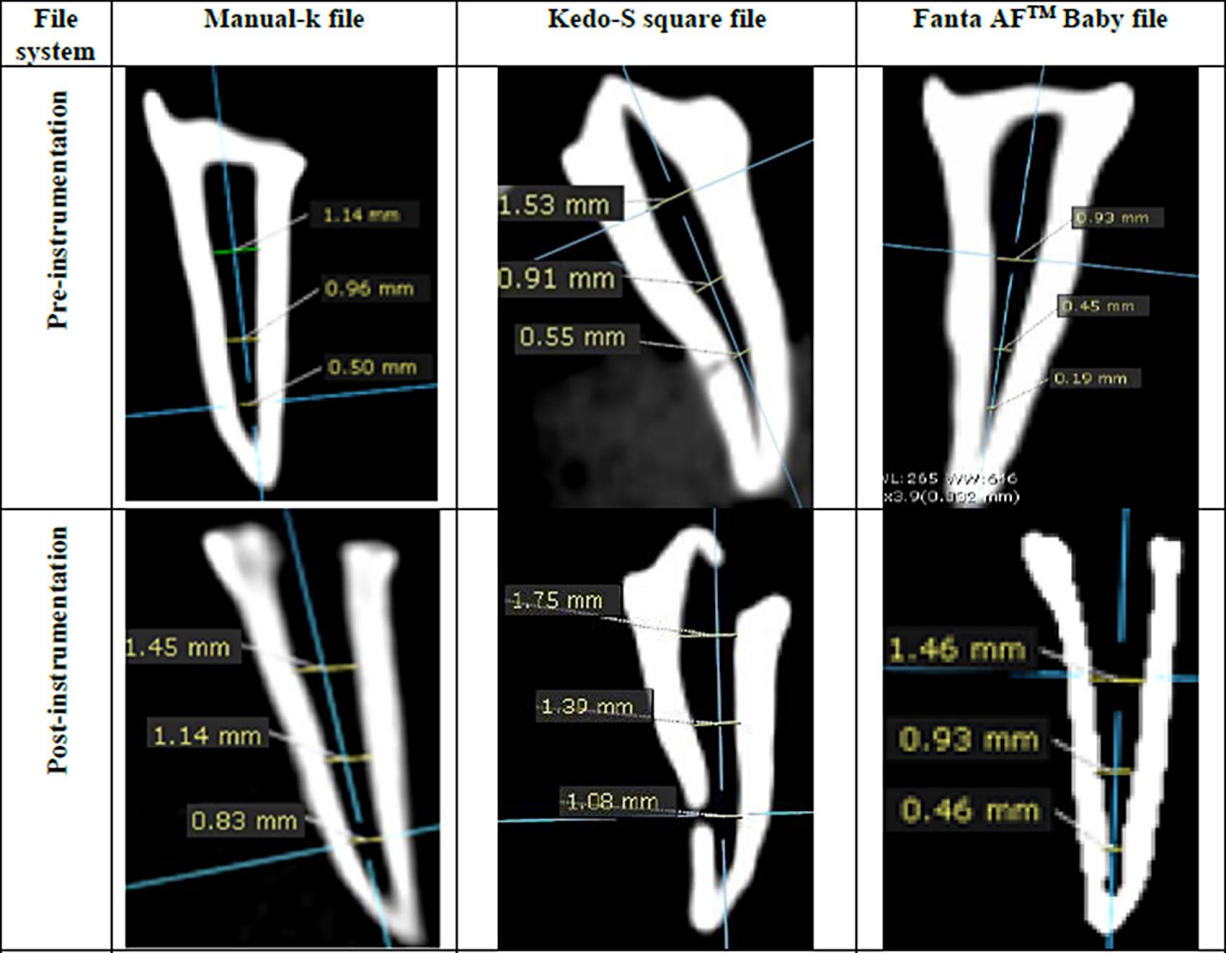
Sagittal cut of CBCT images showing maximum mesiodistal root canal diameter pre- and post-instrumentation with manual K-file, Kedo-S Square file, and Fanta AF^™^ Baby file at cervical, middle, and apical thirds

Concerning centering ability, the highest ratio of teeth with perfect root canal centralization was found in the Kedo-S Square group at different levels with no statistically significant difference between the three groups (*P* > 0.05) at different root canal levels (Table 4). At the cervical third, 60% of the Fanta AF^™^ Baby group and 56% of the K-file group showed total root canal centralization. Regarding the tapering ability, 80% of the Fanta AF^™^ Baby group had good tapered preparation compared to 72% of the Kedo-S Square and 40% of the K-file groups with a statistically significant difference between the three groups (*P* < 0.05) (Table 5; Fig. 2).

**Table 4:** Comparison of centering ability between the file systems at the cervical, middle, and apical levels

Root canal level		Manual K-file (Group I)		Kedo-S Square (Group II)		Fanta AF^™^ Baby (Group III)		*X* ^*2*^	p-value
		n	%	n	%	n	%		
Cervical third	Perfect	14	56.0	18	72.0	15	60.0	1.482	0.477
	Low	11	44.0	7	28.0	10	40.0		
Middle third	Perfect	14	56.0	17	68.0	16	64.0	0.798	0.671
	Low	11	44.0	8	32.0	9	36.0		
Apical third	Perfect	13	52.0	19	76.0	18	72.0	3.720	0.156
	Low	12	48.0	6	24.0	7	28.0		

**Table 5:** Comparison of tapering ability between the file systems

Tapering ability	Manual K-file (Group I)		Kedo-S Square (Group II)		Fanta AF^™^ Baby (Group III)	
	*n*	%	*n*	%	*n*	%
Good	10	40.0	18	72.0	20	80.0
Poor	15	60.0	7	28.0	5	20.0
Chi-square value *(X*^*2*^) = 9.722, p = = 0.008^*^ (significant)						

## Discussion

Biomechanical instrumentation is more challenging in primary teeth due to the presence of accessory canals, ramifications, and intricate torturous root canal anatomy in addition to the child’s negative behavior [26]. Till now, this is the first research that assessed and compared the shaping ability of Kedo-S Square and Fanta AF™ Baby rotary files with manual K-files in primary anterior teeth. These files had been chosen since they exhibit contemporary pediatric file trends. This was in accordance with Mohamed et al. [5]. , who compared the Kedo-S Square, K- & H- hand files in primary canines also, Abd El Fatah et al. [10]. , who compared the Fanta AF™ Baby and Zuanba rotary files with manual K-files in primary mandibular second molars. The Kedo-S Square file is a variable-tapered file that preserves tooth structure more effectively in the mid-root curvature than a fixed-tapered endodontic file [27]. Fanta AF™ Baby files are developed with controlled memory (CM) wires that enable the file to be pre-curved to conform to the canal shape [10].

The present study findings partially rejected the null hypothesis as there was a highly significant statistical difference regards the removed dentin amount on both mesial & distal sides between the three groups at the cervical & apical thirds, whereas a non-significant difference was observed at the middle third (null hypothesis was accepted). These results could be related to variations in the file design, the degree of root canal curvature, instrumentation procedures, and the number of files [28]. This was in contrast with Abd El-Fatah et al. [10] study in which a non-significant statistical difference was found concerning the amount of dentin removed at the coronal & apical thirds among hand K-file, Fanta, and Zuanba groups while a significant difference was present at the middle third (*P* = 0.043). Moreover, in research conducted by Waly et al. [21] comparing K-file with Kedo-S & Pro AF Baby Gold systems, a non-significant difference was found among all groups at all levels regarding the removed dentin thickness.

The Kedo-S Square group in this study removed the least amount of dentin in comparison with the K-file and Fanta AF™ Baby rotary file with high enlargement at the cervical third and restricted apical preparation; this is likely credited to its variable taper (6–8% taper) with the first 5 mm of the file of 6% taper then increase in taper by 7 and 8% [5]. This variable taper design serves to minimize file contact with the root canal while increasing its effectiveness and is more conservative than a fixed taper [29]. The remaining radicular dentin might be the most significant iatrogenic factor that affects the root’s ability to resist fracture; the least remaining dentin thickness of the canal walls should be 0.3 mm to provide adequate protection from occlusal and lateral forces [30]. Particularly in primary teeth, the tooth exfoliates more rapidly when the thickness of the remaining dentin decreases [31]. The present study findings agreed with Mohamed et al. [5] who revealed that the Kedo-S Square file had a minimal quantity of removed dentin contrasted to manual K- & H-files. However, it was disagreed with Güçyetmez et al. [32] who concluded that EndoArt rotary files eliminated more dentin than K-files in the coronal and middle root canal thirds. Using pairwise comparison, a statistically significant difference was found between Kedo-S Square and K-files at different root canal levels in the amount of the removed dentin on both mesial and distal sides. This disagreed with Seema et al. [31] study results in which a non-significant statistical difference between K- & Kedo-S files at the middle & apical thirds of the root canal mesial side; although, the K-file considerably removed a great dentin amount compared to Kedo-S file at the cervical third.

The Kedo-S square group in the present study removed the minimal quantity of dentin in the apical root canal third, with a highly significant statistical difference among the three groups; this could be relevant to the Kedo-S Square dual cross-section as well as, its non-cutting safety end. The Kedo-S Square file has two different cross-sections: the 7 mm coronal part has a teardrop-shaped cross-section while the 5 mm apical part has a triangular cross-Sect. [5]. Such a design produces two-point contact in the coronal section and three-point contact in the apical region, actually resulting in less aggressive preparation & less apical dentin removal, ultimately reducing the lateral perforation risk [33].

In the current study, the greatest amount of dentin removed was noticed in the Fanta AF™ Baby group at the cervical and apical thirds among the study groups; this may be attributed to the constant file taper (0.04 ISO) along the entire file length also, the total number of files used in the instrumentation procedures [30]. A non-significant difference was found between Fanta AF™ Baby and K-file groups at cervical and apical third with a great amount of dentin removed in both groups; this may be linked to the total file number of Fanta AF™ Baby (four files) and K-files (six files) used during the root canal preparation. Also, the constant taper of both files (0.04 ISO of the Fanta AF™ Baby file system and 2% of K-file) along the entire file length [30]. Whereas a significant difference in the middle third was found between the same groups with less amount of dentin removed in the Fanta AF™ Baby group; this may be attributed to less flexible stainless-steel K-files that can force against the dentinal walls laterally and effectively remove more dentin [34], contrasted to controlled memory (CM) wires technology of Fanta AF™ Baby files that easily adapt to diverse canals morphology without straightening [10]. This agreed with Eldemery et al. [30] who found that the K-file group had a higher amount of dentin removed than the Fanta AF™ Baby group at all levels of the root canal with a highly significant difference. Also, Kummer et al. [13] revealed that K-file instrumentation removed more dentin than rotary instrumentation (Hero 642).

These study results revealed that the K-file group removed the highest dentin amount in the middle third among the different groups with a highly significant statistical difference. Moreover, it was noticed that the K-file removed more dentin amount at the apical third on both sides compared to the Kedo-S Square group; this may be related to the stainless-steel K-files’ uncontrollable and violent cutting action [34] in addition to the existence of physiologic or pathologic root resorption in primary teeth. In line with the current study results, Barasuol et al. [35] revealed that ProDesign Logic and Reciproc eliminated minimal dentin from the apical third than K-files using micro-computed tomography.

Regarding root canal transportation in this study, the Kedo-S Square group revealed the highest number of teeth specimens with no transportation at the cervical & apical thirds while, at the middle third, the highest number of no-transportation specimens was present in the Fanta AF™ Baby group with a non-significant statistical difference among different groups. The lower tendency toward apical transportation in the rotary file systems may be credited to its design with a non-cutting tip [5, 10]. This agreed with Güçyetmez et al. [32] study in which a non-significant statistical difference in the apical transportation values was found between K-files & EndoArt rotary files. The highest mesial transportation in the Kedo-S Square group was found in the middle third (40%) in this study while the highest mesial transportation in the Fanta AF™ Baby group was present in the apical third (56%); this may be clarified by the operator’s tendency to do more vigorous preparation on the side opposite to the most favorable support. This agreed with Abd El-Fatah et al. [10] study in which the Fanta AF™ Baby group showed the highest mesial transportation (0.128 mm) at the apical third compared to the K-file and Zuanba rotary system. On the contrary, Haridoss et al. [36]. , revealed that the Kedo-S file showed greater distal displacement (48%) than the Mtwo file (28%) at the cervical root canal level. The highest distal transportation in the present study was noticed in the K-file group at the apical third (48%); this is in contrast with Waly et al. [21] who demonstrated that the K-file had the highest distal transportation (-0.069 mm) at the cervical third compared to the Kedo‑S & Pro AF Baby Gold rotary system.

Concerning centering ability in the current study, the highest ratio of teeth specimens with perfect root canal centralization was found in the Kedo-S Square group with non-significant statistical differences among the three groups at different root canal levels (*P* > 0.05). This may be explained by the heat treatment of Ni-Ti rotary files which leads to an increase in the file’s flexibility, renders higher torsional resistance, adapts the file to the contour of the primary root canal, and aids less straightening during instrumentation [37, 38]. Moreover, the high centering ability of the Fanta AF™ Baby file group could be related to working in a crown-down technique with rotation motion [39]. The decreased centering ability of manual K-file may be linked to the stainless-steel alloy used in its manufacturing which requires more force to bend; this will exert and lead to more cutting on the outer curvature of the canal, which results in eccentricity [40]. This agreed with Waly et al. [21]. , who ascertained that the Kedo‑S rotary file had the highest root canal centralization compared to the K-file & Pro AF Baby Gold rotary system. Also, Selivany & Ahmed’s [41] study revealed that Wave One- Gold reciprocating and one-shape rotary files demonstrated greater centralization than K-files in the root canal apical third. Moreover, Haridoss et al. [36] found that the Kedo-S and Mtwo rotary files preserved the canal centering ability effectively in the middle third than in the coronal & apical thirds with a non-significant difference.

Regarding the tapering ability, 80% of the Fanta AF™ Baby group had good tapered preparation compared to the Kedo-S Square (72%) and K-file (40%) groups with a statistically significant difference among different groups (*P* < 0.05). This can be credited to the design of the Kedo-S Square files, which have a dual cross-section and a variable-taper that corresponds to the anatomy of the root canal of primary teeth, as contrasted with the fixed narrow taper of manual K-files [5]. Also, the Fanta AF™ Baby file with a 4% taper will result in better tapered preparation than that of a 2% tapered hand K-file [5]. Moreover, one of the main factors impacting the root canal taper is the initial canal shape [31]. These results are consistent with Mohamed et al. [5] who found that 75% of the Kedo-S Square group had good tapered preparation compared to 25%, and 15% had good tapered preparation in H-files and K-files respectively. Moreover, it agreed with Ramazani et al. [25]. , who concluded that 75% of the Reciproc group and 81.2% of the Mtwo group had good tapered preparation compared to 31.2% of the K-file group with a statistically significant difference.

The present study’s limitation was to find extracted primary anterior teeth with intact two-thirds of the root length. Further research with large sample size is recommended to evaluate the clinical use of the Kedo-S Square and Fanta AF™ Baby rotary systems.

Based on the present study results, it was concluded that:

- Kedo-S Square rotary file system revealed the least removed dentin compared to Fanta AF™ Baby rotary files and hand K-files at all levels except at the middle third, with significant differences between the three groups.
- The Kedo-S Square group had perfect root canal centralization at different levels.
- Manual K-file demonstrated more dentin removal and more canal transportation compared to the other two groups.
- The Kedo-S Square rotary file was preferable to the Fanta AF^™^ Baby and manual K-file regarding dentin thickness preservation, canal transportation & centering ability after root canal instrumentation.

### Bullet points

- Biomechanical preparation performs a significant role in the success of primary teeth pulpectomy.
- Kedo-S Square & Fanta rotary files were used to overcome shortcomings of manual preparation.
- CBCT helps non-intrusive reproducible assessment of root canal configuration changes.

## Data Availability

The data sources utilised and interpreted during the present research are accessible upon reasonable request from the corresponding author.
